# Supplementary material for: Unveiling the sensory and interneuronal pathways of the neuroendocrine connectome in Drosophila
Source: eLife. 2021 Jun 4;10:e65745. doi: 10.7554/eLife.65745 (PMC8177888; doi:10.7554/eLife.65745)
Supplement: Figure 2—source data 1. [file elife-65745-fig2-data1.docx]

**Figure 2 - source data 1. *Drosophila* RPNs in pars intercerebralis (PI).**

| **RPNs** | **Synonyms** | **Neuropeptides expressed** | **Neuropeptide/neurotransmitter receptors expressed** | **RPN Functions** |
| --- | --- | --- | --- | --- |
| IPCs | - mNSCs - dILPs - CC-PI | - Ilp1, 2, 3, 5 (Insulin-like peptide 1, 2, 3, 5) (Brogiolo et al., 2001) - Dsk (Drosulfakinin) (Söderberg et al., 2012) | - Oamb (Octopamine receptor in mushroom bodies) (Crocker et al. 2010) - GABA-B-R2 (metabotropic GABA-B receptor subtype 2) (Enell et al., 2010) - TkR99D (Tachykinin-like receptor at 99D) (Birse et al., 2011) - slo (slowpoke), Slob (Slowpoke binding protein) (Sheldon et al., 2011) - CrzR (Corazonin receptor) (Kapan et al., 2012) - 5-HT1A (serotonin receptor 1A) (Luo et al., 2012) - AstA-R2 (Allatostatin A receptor 2) (Hentze et al., 2015) - CCHa2-R (CCHamide-2 receptor) (Sano et al., 2015) - PK2-R1 (Pyrokinin 2 receptor 1) (Schlegel et al., 2016) - Lkr (Leucokinin receptor) (Zandawala et al., 2018) - AstA-R1, -R2 (Allatostatin A receptor 1, 2), AstC-R2 (Allatostatin C receptor 2), Octβ1R (Octopamine β1 receptor) (Cocanougher et al., 2019) - sNPF-R (short neuropeptide F receptor) (Oh et al., 2019) | - Diapause (Tatar and Yin, 2001) - Fecundity and lifespan (Broughton et al., 2005) - Sugar metabolism (Broughton et al., 2005 and 2008) - Stress resistance (Broughton et al., 2005; Karpac et al., 2009; Grönke et al., 2010; Zandawala et al., 2018) - Food intake regulation (Wu et al., 2005; Söderberg et al., 2012) - Locomotion (Belgacem and Martin, 2006; Jones et al., 2009) - Sleep (Crocker et al., 2010) - Development and growth delay (Grönke et al. 2010) - Sensitivity to food odors (Root et al., 2011) - Starvation resistance (Kapan et al., 2012) - Clock circuit (Cong et al., 2015) - Food preference (Semaniuk et al., 2018) |
| DMS | - mNSCs | - Ms (Myosuppressin) (McCormick and Nichols, 1993) | - PK2-R1 (Pyrokinin 2 receptor 1) (Schlegel et al., 2016) | - Myoinhibitory on visceral, crop and gut muscles (Johnson et al., 2000; Merte and Nichols, 2002; Dickerson et al., 2012) - Locomotion velocity (Kiss et al., 2013) - Triger for eclosion (Ruf et al., 2017) - CO_2_ detection circuit **(this study)** |
| DH44 | - mNSCs | - Dh44 (Diuretic hormone 44) (Cabrero et al., 2002) - Nplp2, 3 (Neuropeptide-like precursor 2, 3) (Cavanaugh et al., 2014) - Ilp2 (Insulin-like peptide 2) (Ohhara et al., 2018) | - PK2-R1 (Pyrokinin 2 receptor 1) (Schlegel et al., 2016) - Lkr (Leucokinin receptor) (Cannell et al., 2016) | - Diuretic function (Cabrero et al., 2002; Hector et al., 2009; Dus et al., 2015) - Food search and feeding (Söderberg et al., 2012) - Rest:activity rhythms (Cavanaugh et al., 2014) - Postingestive glucose sensor (Dus et al., 2015) - Sperm ejection and storage (Lee et al., 2015) - Stress regulation (Cannell et al., 2016) - Postingestive amino acid sensor (Yang et al., 2018) - CO_2_ detection circuit **(this study)** |

**Figure 2 - source data 1. *Drosophila* RPNs in pars lateralis (PL).**

| **RPNs** | **Synonyms** | **Neuropeptides**  **expressed** | **Neuropeptide/neurotransmitter receptors**  **expressed** | **RPN Functions** |
| --- | --- | --- | --- | --- |
| CRZ | - DLP - DN1 - CC-PL-1 - CN neurons | - Crz (Corazonin) (Choi et al., 2005) - Proc (Proctolin) (Isaac et al., 2004) - sNPF (short neuropeptide F) (Nässel et al., 2008; Kapan et al., 2012) - Dsk (Drosulfakinin) (Söderberg et al., 2012) | - Dh44-R1 (Diuretic hormone 44 receptor 1), Dh31-R (Diuretic hormone 31 receptor) (Johnson et al., 2005) - AstA-R2 (Allatostatin A receptor 2) (Johnson et al., 2005; Veenstra, 2009) - Oamb (Octopamine receptor in mushroom bodies) (Imura et al., 2020) | - Initiation of ecdysis (Kim et al., 2004) - Stress regulation (Veenstra, 2009; Kapan et al., 2012; Kubrak et al., 2016; Zhao et al., 2010) - Fructose sensor (Miyamoto et al., 2012) - Feeding regulation (Miyamoto et al., 2012; Hergarden et al., 2012) - Ethanol tolerance (Sha et al., 2014) - Egg laying (Gospocic et al., 2017) - Growth regulation (via PTTH) (Imura et al., 2020) - Glucose sensing/homeostasis (Oh et al., 2019) - CO_2_ detection circuit **(this study)** |
| ITP | - ipc-1 - ALK - CC-PL-2 | - ITP (Ion transport peptide) (Dircksen et al., 2008) - Lk (Leucokinin) (de Haro et al., 2010) - sNPF (short neuropeptide F), Tk (Tachykinin) (Kahsai et al., 2010) | - Unknown | - Anti-diuretic, water and ion homeostasis (Kahsai et al., 2010; Gáliková et al., 2018) - Food search and feeding (Gáliková et al., 2018) |
| PTTH | - PG-LP | - Ptth (Prothoracicotropic hormone) (McBrayer et al., 2007) | - CrzR (Corazonin receptor) (Imura et al., 2020) | - Regulation of ecdysone production (McBrayer et al., 2007) - Promotes light avoidance at end of larval stage (Yamanaka et al., 2013) - Circadian rhythmicity of eclosion (Selcho et al., 2017) - Metamorphosis onset, reproductive capacity (Shimell et al., 2018) - Growth (Colombani et al., 2012) |
| CA-LP |  | - FMRFa (FMRFamide) (Hartenstein, 2006) - Burs (Bursicon) **(this study)** | - Unknown | - Unknown |

**Figure 2 - source data 1. *Drosophila* RPNs in subesophageal zone (SEZ) or protocerebrum.**

| **RPNs** | **Synonyms** | **Neuropeptides**  **expressed** | **Neuropeptide/neurotransmitter receptors**  **expressed** | **RPN Functions** |
| --- | --- | --- | --- | --- |
| HugRG | - CC-MS1 | - Hug (Hugin) (Melcher and Pankratz, 2005; Schlegel et al., 2016) | - Unknown | - Unknown function for this subpopulation of Hugin cells |
| CAPA | - CC-MS2 | - Capa (Capability) (Kean et al., 2002; Wegener et al., 2006) | - Unknown | - Unknown |
| EH | - VM neurons | - Eh (Eclosion hormone) (Hodoroyski et al., 1993) | - ETHR (Kim et al., 2006) | - Onset of ecdysis behavior (Truman, 1992; Baker et al., 1999) - Coordination of eclosion (McNabb et al., 1997) - Tracheal filling (Baker et al., 1999) - Pre-ecdysis behavior (Krüger et al., 2015) |

Much of the information presented in Figure 2 - source data 1 is comprehensively shown and summarized in Siegmund and Korge (2001), Nässel et al. (2008), Nässel and Winther (2010), Nässel et al. (2013) and Nässel and Zandawala (2019).

**References**

Baker JD, McNabb SL, Truman JW. 1999. The hormonal coordination of behavior and physiology at adult ecdysis in Drosophila melanogaster. *J Exp Biol* **202**:3037–3048.

[Belgacem YH, Martin J-R. 2006. Disruption of insulin pathways alters trehalose level and abolishes sexual dimorphism in locomotor activity in Drosophila. *J Neurobiol* **66**:19–32.](http://paperpile.com/b/mo8Ctq/IbtZ)

[Birse RT, Soderberg JAE, Luo J, Winther AME, Nassel DR. 2011. Regulation of insulin-producing cells in the adult Drosophila brain via the tachykinin peptide receptor DTKR. *Journal of Experimental Biology*. doi:](http://paperpile.com/b/mo8Ctq/Lpzs)[10.1242/jeb.062091](http://dx.doi.org/10.1242/jeb.062091)

[Brogiolo W, Stocker H, Ikeya T, Rintelen F, Fernandez R, Hafen E. 2001. An evolutionarily conserved function of the Drosophila insulin receptor and insulin-like peptides in growth control. *Curr Biol* **11**:213–221.](http://paperpile.com/b/mo8Ctq/L5N7)

[Broughton S, Alic N, Slack C, Bass T, Ikeya T, Vinti G, Tommasi AM, Driege Y, Hafen E, Partridge L. 2008. Reduction of DILP2 in Drosophila triages a metabolic phenotype from lifespan revealing redundancy and compensation among DILPs. *PLoS One* **3**:e3721.](http://paperpile.com/b/mo8Ctq/aDa3)

[Broughton SJ, Piper MDW, Ikeya T, Bass TM, Jacobson J, Driege Y, Martinez P, Hafen E, Withers DJ, Leevers SJ, Partridge L. 2005. Longer lifespan, altered metabolism, and stress resistance in Drosophila from ablation of cells making insulin-like ligands. *Proc Natl Acad Sci U S A* **102**:3105–3110.](http://paperpile.com/b/mo8Ctq/6Kc8)

[Cabrero P, Radford JC, Broderick KE, Costes L, Veenstra JA, Spana EP, Davies SA, Dow JAT. 2002. The Dh gene of Drosophila melanogaster encodes a diuretic peptide that acts through cyclic AMP. *J Exp Biol* **205**:3799–3807.](http://paperpile.com/b/mo8Ctq/nVpK)

Cannell E, Dornan AJ, Halberg KA, Terhzaz S, Dow JAT, Davies S-A. 2016. The corticotropin-releasing factor-like diuretic hormone 44 (DH44) and kinin neuropeptides modulate desiccation and starvation tolerance in Drosophila melanogaster. *Peptides*. doi:[10.1016/j.peptides.2016.02.004](http://dx.doi.org/10.1016/j.peptides.2016.02.004)

[Cavanaugh DJ, Geratowski JD, Wooltorton JRA, Spaethling JM, Hector CE, Zheng X, Johnson EC, Eberwine JH, Sehgal A. 2014. Identification of a Circadian Output Circuit for Rest:Activity Rhythms in Drosophila. *Cell*. doi:](http://paperpile.com/b/mo8Ctq/fNB7)[10.1016/j.cell.2014.02.024](http://dx.doi.org/10.1016/j.cell.2014.02.024)

[Choi YJ, Lee G, Hall JC, Park JH. 2005. Comparative analysis of Corazonin-encoding genes (Crz’s) in Drosophila species and functional insights into Crz-expressing neurons. *J Comp Neurol* **482**:372–385.](http://paperpile.com/b/mo8Ctq/wxNr)

[Cocanougher BT, Wittenbach JD, Long XS, Kohn AB, Norekian TP, Yan J, Colonell J, Masson J-B, Truman JW, Cardona A, Turaga SC, Singer RH, Moroz LL, Zlatic M. 2019. Comparative single-cell transcriptomics of complete insect nervous systems. *bioRxiv*. doi:](http://paperpile.com/b/mo8Ctq/kV60)[10.1101/785931](http://dx.doi.org/10.1101/785931)

[Colombani J, Andersen DS, Léopold P. 2012. Secreted peptide Dilp8 coordinates Drosophila tissue growth with developmental timing. *Science* **336**:582–585.](http://paperpile.com/b/mo8Ctq/iyDk)

[Cong X, Wang H, Liu Z, He C, An C, Zhao Z. 2015. Regulation of Sleep by Insulin-like Peptide System in Drosophila melanogaster. *Sleep* **38**:1075–1083.](http://paperpile.com/b/mo8Ctq/80g3)

[Crocker A, Shahidullah M, Levitan IB, Sehgal A. 2010. Identification of a neural circuit that underlies the effects of octopamine on sleep:wake behavior. *Neuron* **65**:670–681.](http://paperpile.com/b/mo8Ctq/lSnw)

[de Haro M, Al-Ramahi I, Benito-Sipos J, López-Arias B, Dorado B, Veenstra JA, Herrero P. 2010. Detailed analysis of leucokinin-expressing neurons and their candidate functions in the Drosophila nervous system. *Cell Tissue Res* **339**:321–336.](http://paperpile.com/b/mo8Ctq/BOFl)

[Dickerson M, McCormick J, Mispelon M, Paisley K, Nichols R. 2012. Structure-activity and immunochemical data provide evidence of developmental- and tissue-specific myosuppressin signaling. *Peptides* **36**:272–279.](http://paperpile.com/b/mo8Ctq/11CJ)

[Dircksen H, Tesfai LK, Albus C, Nässel DR. 2008. Ion transport peptide splice forms in central and peripheral neurons throughout postembryogenesis of Drosophila melanogaster. *J Comp Neurol* **509**:23–41.](http://paperpile.com/b/mo8Ctq/Zfam)

[Dus M, Lai JS-Y, Gunapala KM, Min S, Tayler TD, Hergarden AC, Geraud E, Joseph CM, Suh GSB. 2015. Nutrient Sensor in the Brain Directs the Action of the Brain-Gut Axis in Drosophila. *Neuron* **87**:139–151.](http://paperpile.com/b/mo8Ctq/HalQ)

[Enell LE, Kapan N, Söderberg JAE, Kahsai L, Nässel DR. 2010. Insulin signaling, lifespan and stress resistance are modulated by metabotropic GABA receptors on insulin producing cells in the brain of Drosophila. *PLoS One* **5**:e15780.](http://paperpile.com/b/mo8Ctq/YTyo)

[Gáliková M, Dircksen H, Nässel DR. 2018. The thirsty fly: Ion transport peptide (ITP) is a novel endocrine regulator of water homeostasis in Drosophila. *PLoS Genet* **14**:e1007618.](http://paperpile.com/b/mo8Ctq/tvNV)

[Gospocic J, Shields EJ, Glastad KM, Lin Y, Penick CA, Yan H, Mikheyev AS, Linksvayer TA, Garcia BA, Berger SL, Liebig J, Reinberg D, Bonasio R. 2017. The Neuropeptide Corazonin Controls Social Behavior and Caste Identity in Ants. *Cell* **170**:748–759.e12.](http://paperpile.com/b/mo8Ctq/pAJC)

[Grönke S, Clarke D-F, Broughton S, Andrews TD, Partridge L. 2010. Molecular evolution and functional characterization of Drosophila insulin-like peptides. *PLoS Genet* **6**:e1000857.](http://paperpile.com/b/mo8Ctq/LFaP)

[Hartenstein V. 2006. The neuroendocrine system of invertebrates: a developmental and evolutionary perspective. *J Endocrinol* **190**:555–570.](http://paperpile.com/b/mo8Ctq/LA08)

[Hector CE, Bretz CA, Zhao Y, Johnson EC. 2009. Functional differences between two CRF-related diuretic hormone receptors in Drosophila. *J Exp Biol* **212**:3142–3147.](http://paperpile.com/b/mo8Ctq/bvsT)

[Hentze JL, Carlsson MA, Kondo S, Nässel DR, Rewitz KF. 2015. The Neuropeptide Allatostatin A Regulates Metabolism and Feeding Decisions in Drosophila. *Sci Rep* **5**:11680.](http://paperpile.com/b/mo8Ctq/kfvz)

[Hergarden AC, Tayler TD, Anderson DJ. 2012. Allatostatin-A neurons inhibit feeding behavior in adult Drosophila. *Proc Natl Acad Sci U S A* **109**:3967–3972.](http://paperpile.com/b/mo8Ctq/Z1Eb)

[Horodyski FM, Ewer J, Riddiford LM, Truman JW. 1993. Isolation, characterization and expression of the eclosion hormone gene of Drosophila melanogaster. *Eur J Biochem* **215**:221–228.](http://paperpile.com/b/mo8Ctq/jRLQ)

[Imura E, Shimada-Niwa Y, Nishimura T, Hückesfeld S, Schlegel P, Ohhara Y, Kondo S, Tanimoto H, Cardona A, Pankratz MJ, Niwa R. 2020. The Corazonin-PTTH Neuronal Axis Controls Systemic Body Growth by Regulating Basal Ecdysteroid Biosynthesis in Drosophila melanogaster. *Curr Biol* **30**:2156–2165.e5.](http://paperpile.com/b/mo8Ctq/gwQO)

[Isaac RE, Taylor CA, Hamasaka Y, Nässel DR, Shirras AD. 2004. Proctolin in the post-genomic era: new insights and challenges. *Invert Neurosci* **5**:51–64.](http://paperpile.com/b/mo8Ctq/x51m)

[Johnson EC, Shafer OT, Trigg JS, Park J, Schooley DA, Dow JA, Taghert PH. 2005. A novel diuretic hormone receptor in Drosophila: evidence for conservation of CGRP signaling. *J Exp Biol* **208**:1239–1246.](http://paperpile.com/b/mo8Ctq/QJzn)

[Johnson E, Ringo J, Dowse H. 2000. Native and heterologous neuropeptides are cardioactive in Drosophila melanogaster. *J Insect Physiol* **46**:1229–1236.](http://paperpile.com/b/mo8Ctq/7ioe)

[Jones MA, Gargano JW, Rhodenizer D, Martin I, Bhandari P, Grotewiel M. 2009. A forward genetic screen in Drosophila implicates insulin signaling in age-related locomotor impairment. *Exp Gerontol* **44**:532–540.](http://paperpile.com/b/mo8Ctq/zBnZ)

[Kahsai L, Kapan N, Dircksen H, Winther AME, Nässel DR. 2010. Metabolic stress responses in Drosophila are modulated by brain neurosecretory cells that produce multiple neuropeptides. *PLoS One* **5**:e11480.](http://paperpile.com/b/mo8Ctq/M1Db)

[Kapan N, Lushchak OV, Luo J, Nässel DR. 2012. Identified peptidergic neurons in the Drosophila brain regulate insulin-producing cells, stress responses and metabolism by coexpressed short neuropeptide F and corazonin. *Cell Mol Life Sci* **69**:4051–4066.](http://paperpile.com/b/mo8Ctq/XUFC)

[Karpac J, Hull-Thompson J, Falleur M, Jasper H. 2009. JNK signaling in insulin-producing cells is required for adaptive responses to stress in Drosophila. *Aging Cell* **8**:288–295.](http://paperpile.com/b/mo8Ctq/vj1C)

[Kean L, Cazenave W, Costes L, Broderick KE, Graham S, Pollock VP, Davies SA, Veenstra JA, Dow JAT. 2002. Two nitridergic peptides are encoded by the gene capability in Drosophila melanogaster. *Am J Physiol Regul Integr Comp Physiol* **282**:R1297–307.](http://paperpile.com/b/mo8Ctq/xHqO)

[Kim Y-J, Spalovská-Valachová I, Cho K-H, Zitnanova I, Park Y, Adams ME, Zitnan D. 2004. Corazonin receptor signaling in ecdysis initiation. *Proc Natl Acad Sci U S A* **101**:6704–6709.](http://paperpile.com/b/mo8Ctq/agq3)

[Kim Y-J, Zitnan D, Galizia CG, Cho K-H, Adams ME. 2006. A command chemical triggers an innate behavior by sequential activation of multiple peptidergic ensembles. *Curr Biol* **16**:1395–1407.](http://paperpile.com/b/mo8Ctq/qKdY)

[Kiss B, Szlanka T, Zvara Á, Žurovec M, Sery M, Kakaš Š, Ramasz B, Hegedűs Z, Lukacsovich T, Puskás L, Fónagy A, Kiss I. 2013. Selective elimination/RNAi silencing of FMRF-related peptides and their receptors decreases the locomotor activity in Drosophila melanogaster. *Gen Comp Endocrinol* **191**:137–145.](http://paperpile.com/b/mo8Ctq/qyUf)

[Krüger E, Mena W, Lahr EC, Johnson EC, Ewer J. 2015. Genetic analysis of Eclosion hormone action during Drosophila larval ecdysis. *Development* **142**:4279–4287.](http://paperpile.com/b/mo8Ctq/n7l1)

[Kubrak OI, Lushchak OV, Zandawala M, Nässel DR. 2016. Systemic corazonin signalling modulates stress responses and metabolism in Drosophila. *Open Biol* **6**. doi:](http://paperpile.com/b/mo8Ctq/8q2h)[10.1098/rsob.160152](http://dx.doi.org/10.1098/rsob.160152)

[Lee K-M, Daubnerová I, Isaac RE, Zhang C, Choi S, Chung J, Kim Y-J. 2015. A neuronal pathway that controls sperm ejection and storage in female Drosophila. *Curr Biol* **25**:790–797.](http://paperpile.com/b/mo8Ctq/vaWG)

[Luo J, Becnel J, Nichols CD, Nässel DR. 2012. Insulin-producing cells in the brain of adult Drosophila are regulated by the serotonin 5-HT1A receptor. *Cell Mol Life Sci* **69**:471–484.](http://paperpile.com/b/mo8Ctq/ihsP)

[McBrayer Z, Ono H, Shimell M, Parvy J-P, Beckstead RB, Warren JT, Thummel CS, Dauphin-Villemant C, Gilbert LI, O’Connor MB. 2007. Prothoracicotropic hormone regulates developmental timing and body size in Drosophila. *Dev Cell* **13**:857–871.](http://paperpile.com/b/mo8Ctq/yEWb)

[McCormick J, Nichols R. 1993. Spatial and temporal expression identify dromyosuppressin as a brain-gut peptide in Drosophila melanogaster. *J Comp Neurol* **338**:278–288.](http://paperpile.com/b/mo8Ctq/aYGh)

[McNabb SL, Baker JD, Agapite J, Steller H, Riddiford LM, Truman JW. 1997. Disruption of a behavioral sequence by targeted death of peptidergic neurons in Drosophila. *Neuron* **19**:813–823.](http://paperpile.com/b/mo8Ctq/ghWM)

[Melcher C, Pankratz MJ. 2005. Candidate gustatory interneurons modulating feeding behavior in the Drosophila brain. *PLoS Biol* **3**:e305.](http://paperpile.com/b/mo8Ctq/2E0S)

[Merte J, Nichols R. 2002. Drosophila melanogaster myotropins have unique functions and signaling pathways. *Peptides*. doi:](http://paperpile.com/b/mo8Ctq/DQ6E)[10.1016/s0196-9781(01)00670-2](http://dx.doi.org/10.1016/s0196-9781(01)00670-2)

[Miyamoto T, Slone J, Song X, Amrein H. 2012. A Fructose Receptor Functions as a Nutrient Sensor in the Drosophila Brain. *Cell*. doi:](http://paperpile.com/b/mo8Ctq/O1Yw)[10.1016/j.cell.2012.10.024](http://dx.doi.org/10.1016/j.cell.2012.10.024)

[Nässel DR, Enell LE, Santos JG, Wegener C, Johard HAD. 2008. A large population of diverse neurons in the Drosophila central nervous system expresses short neuropeptide F, suggesting multiple distributed peptide functions. *BMC Neurosci* **9**:90.](http://paperpile.com/b/mo8Ctq/DEjn)

[Nässel DR, Kubrak OI, Liu Y, Luo J, Lushchak OV. 2013. Factors that regulate insulin producing cells and their output in Drosophila. *Front Physiol* **4**:252.](http://paperpile.com/b/mo8Ctq/zBZR)

[Nässel DR, Winther AME. 2010. Drosophila neuropeptides in regulation of physiology and behavior. *Prog Neurobiol* **92**:42–104.](http://paperpile.com/b/mo8Ctq/hkQB)

[Nässel DR, Zandawala M. 2019. Recent advances in neuropeptide signaling in Drosophila, from genes to physiology and behavior. *Prog Neurobiol* **179**:101607.](http://paperpile.com/b/mo8Ctq/RCdl)

[Ohhara Y, Kobayashi S, Yamakawa-Kobayashi K, Yamanaka N. 2018. Adult-specific insulin-producing neurons in Drosophila melanogaster. *J Comp Neurol* **526**:1351–1367.](http://paperpile.com/b/mo8Ctq/1MBR)

[Oh Y, Lai JS-Y, Mills HJ, Erdjument-Bromage H, Giammarinaro B, Saadipour K, Wang JG, Abu F, Neubert TA, Suh GSB. 2019. A glucose-sensing neuron pair regulates insulin and glucagon in Drosophila. *Nature* **574**:559–564.](http://paperpile.com/b/mo8Ctq/vzkS)

[Root CM, Ko KI, Jafari A, Wang JW. 2011. Presynaptic facilitation by neuropeptide signaling mediates odor-driven food search. *Cell* **145**:133–144.](http://paperpile.com/b/mo8Ctq/xbcI)

[Ruf F, Fraunholz M, Öchsner K, Kaderschabek J, Wegener C. 2017. WEclMon - A simple and robust camera-based system to monitor Drosophila eclosion under optogenetic manipulation and natural conditions. *PLoS One* **12**:e0180238.](http://paperpile.com/b/mo8Ctq/tUDw)

[Sano H, Nakamura A, Texada MJ, Truman JW, Ishimoto H, Kamikouchi A, Nibu Y, Kume K, Ida T, Kojima M. 2015. The Nutrient-Responsive Hormone CCHamide-2 Controls Growth by Regulating Insulin-like Peptides in the Brain of Drosophila melanogaster. *PLoS Genet* **11**:e1005209.](http://paperpile.com/b/mo8Ctq/85Tf)

[Schlegel P, Texada MJ, Miroschnikow A, Schoofs A, Hückesfeld S, Peters M, Schneider-Mizell CM, Lacin H, Li F, Fetter RD, Truman JW, Cardona A, Pankratz MJ. 2016. Synaptic transmission parallels neuromodulation in a central food-intake circuit. *Elife* **5**. doi:](http://paperpile.com/b/mo8Ctq/txFE)[10.7554/eLife.16799](http://dx.doi.org/10.7554/eLife.16799)

[Selcho M, Millán C, Palacios-Muñoz A, Ruf F, Ubillo L, Chen J, Bergmann G, Ito C, Silva V, Wegener C, Ewer J. 2017. Central and peripheral clocks are coupled by a neuropeptide pathway in Drosophila. *Nat Commun* **8**:15563.](http://paperpile.com/b/mo8Ctq/6Rki)

[Semaniuk UV, Gospodaryov DV, Feden’ko KM, Yurkevych IS, Vaiserman AM, Storey KB, Simpson SJ, Lushchak O. 2018. Insulin-Like Peptides Regulate Feeding Preference and Metabolism in Drosophila. *Frontiers in Physiology*. doi:](http://paperpile.com/b/mo8Ctq/drvS)[10.3389/fphys.2018.01083](http://dx.doi.org/10.3389/fphys.2018.01083)

[Sha K, Choi S-H, Im J, Lee GG, Loeffler F, Park JH. 2014. Regulation of ethanol-related behavior and ethanol metabolism by the Corazonin neurons and Corazonin receptor in Drosophila melanogaster. *PLoS One* **9**:e87062.](http://paperpile.com/b/mo8Ctq/x6H9)

[Sheldon AL, Zhang J, Fei H, Levitan IB. 2011. SLOB, a SLOWPOKE Channel Binding Protein, Regulates Insulin Pathway Signaling and Metabolism in Drosophila. *PLoS ONE*. doi:](http://paperpile.com/b/mo8Ctq/m9ky)[10.1371/journal.pone.0023343](http://dx.doi.org/10.1371/journal.pone.0023343)

[Shimell M, Pan X, Martin FA, Ghosh AC, Leopold P, O’Connor MB, Romero NM. 2018. Prothoracicotropic hormone modulates environmental adaptive plasticity through the control of developmental timing. *Development* **145**. doi:](http://paperpile.com/b/mo8Ctq/qco9)[10.1242/dev.159699](http://dx.doi.org/10.1242/dev.159699)

[Siegmund T, Korge G. 2001. Innervation of the ring gland of Drosophila melanogaster. *J Comp Neurol* **431**:481–491.](http://paperpile.com/b/mo8Ctq/9YPp)

[Söderberg JAE, Carlsson MA, Nässel DR. 2012. Insulin-Producing Cells in the Drosophila Brain also Express Satiety-Inducing Cholecystokinin-Like Peptide, Drosulfakinin. *Front Endocrinol*  **3**:109.](http://paperpile.com/b/mo8Ctq/Tifg)

[Tatar M, Yin C. 2001. Slow aging during insect reproductive diapause: why butterflies, grasshoppers and flies are like worms. *Exp Gerontol* **36**:723–738.](http://paperpile.com/b/mo8Ctq/be5A)

[Truman JW. 1992. Chapter 30 The eclosion hormone system of insects. *Progress in Brain Research*. doi:](http://paperpile.com/b/mo8Ctq/sX3Y)[10.1016/s0079-6123(08)61189-9](http://dx.doi.org/10.1016/s0079-6123(08)61189-9)

[Veenstra JA. 2009. Does corazonin signal nutritional stress in insects? *Insect Biochem Mol Biol* **39**:755–762.](http://paperpile.com/b/mo8Ctq/WCTu)

[Wegener C, Reinl T, Jänsch L, Predel R. 2006. Direct mass spectrometric peptide profiling and fragmentation of larval peptide hormone release sites in Drosophila melanogaster reveals tagma-specific peptide expression and differential processing. *J Neurochem* **96**:1362–1374.](http://paperpile.com/b/mo8Ctq/xymD)

[Wu Q, Zhang Y, Xu J, Shen P. 2005. Regulation of hunger-driven behaviors by neural ribosomal S6 kinase in Drosophila. *Proc Natl Acad Sci U S A* **102**:13289–13294.](http://paperpile.com/b/mo8Ctq/lEiy)

[Yamanaka N, Romero NM, Martin FA, Rewitz KF, Sun M, O’Connor MB, Léopold P. 2013. Neuroendocrine control of Drosophila larval light preference. *Science* **341**:1113–1116.](http://paperpile.com/b/mo8Ctq/emic)

[Yang Z, Huang R, Fu X, Wang G, Qi W, Mao D, Shi Z, Shen WL, Wang L. 2018. A post-ingestive amino acid sensor promotes food consumption in Drosophila. *Cell Res* **28**:1013–1025.](http://paperpile.com/b/mo8Ctq/vv1i)

[Zandawala M, Yurgel ME, Texada MJ, Liao S, Rewitz KF, Keene AC, Nässel DR. 2018. Modulation of Drosophila post-feeding physiology and behavior by the neuropeptide leucokinin. *PLoS Genet* **14**:e1007767.](http://paperpile.com/b/mo8Ctq/6vnP)

[Zhao Y, Bretz CA, Hawksworth SA, Hirsh J, Johnson EC. 2010. Corazonin neurons function in sexually dimorphic circuitry that shape behavioral responses to stress in Drosophila. *PLoS One* **5**:e9141.](http://paperpile.com/b/mo8Ctq/1K1U)
