# Supplementary material for: Unveiling the sensory and interneuronal pathways of the neuroendocrine connectome in Drosophila
Source: eLife. 2021 Jun 4;10:e65745. doi: 10.7554/eLife.65745 (PMC8177888; doi:10.7554/eLife.65745)
Supplement: Supplementary file 1. — Green to red ratios in CaMPARI measurements. For experiments and use of statistical tests, see Materials and methods. [file elife-65745-supp1.docx]

Supplementary file 1

**Statistics table.** Green to red ratios in CaMPARI measurements. For experiments and use of statistical tests, see Materials and Methods

|  | **N** | **mean** | **median** | **SD** | **SEM** | **test used** | **p-value** |
| --- | --- | --- | --- | --- | --- | --- | --- |
| **Figure 3 – figure supplement 2 B** | | | | | | | |
| Air | 10 | 0.8008 | 0.7748 | 0.1560 | 0.04934 |  |  |
| CO2 | 10 | 2.201 | 2.126 | 0.5680 | 0.1796 | Mann-Whitney-Rank-Sum | <0.0001 |
| **Figure 3 - figure supplement 2 C** | | | | | | | |
| 0% CO2 | 15 | 1.174 | 1.128 | 0.2080 | 0.05370 |  |  |
| 10% CO2 | 15 | 1.457 | 1.393 | 0.1576 | 0.04068 | Mann-Whitney-Rank-Sum | 0.0002 |
| 20% CO2 | 15 | 1.906 | 1.807 | 0.4400 | 0.1136 | Mann-Whitney-Rank-Sum | 0.0017 |
| **Figure 4 B** | | | | | | | |
| DMS Air | 10 | 0.4649 | 0.4019 | 0.2093 | 0.06619 |  |  |
| DMS CO2 | 11 | 0.7254 | 0.7155 | 0.3237 | 0.09759 | Mann-Whitney-Rank-Sum | 0.403 |
| IPCs Air | 10 | 0.2096 | 0.2060 | 0.04753 | 0.01503 |  |  |
| IPCs CO2 | 10 | 0.2513 | 0.2376 | 0.06013 | 0.01902 | Mann-Whitney-Rank-Sum | 0.2176 |
| DH44 Air | 11 | 0.3854 | 0.3300 | 0.1412 | 0.04257 |  |  |
| DH44 CO2 | 10 | 0.6412 | 0.6180 | 0.2816 | 0.08906 | Mann-Whitney-Rank-Sum | 0.0079 |
| CRZ Air | 10 | 3.819 | 2.156 | 3.962 | 1.253 |  |  |
| CRZ CO2 | 10 | 6.709 | 6.555 | 3.695 | 1.169 | Mann-Whitney-Rank-Sum | 0.0355 |
| LK Air | 11 | 0.8000 | 0.8409 | 0.1482 | 0.04470 |  |  |
| LK CO2 | 11 | 0.6627 | 0.6676 | 0.1592 | 0.04799 | Mann-Whitney-Rank-Sum | 0.0759 |
| PTTH Air | 10 | 1.363 | 1.377 | 0.6519 | 0.2061 |  |  |
| PTTH CO2 | 10 | 1.064 | 1.038 | 0.1999 | 0.06323 | Mann-Whitney-Rank-Sum | 0.4686 |
| HugRG Air | 10 | 0.2320 | 0.2228 | 0.06208 | 0.01963 |  |  |
| HugRG CO2 | 10 | 0.2497 | 0.2438 | 0.03405 | 0.01077 | Mann-Whitney-Rank-Sum | 0.0535 |
| CAPA Air | 11 | 2.331 | 2.280 | 1.064 | 0.3209 |  |  |
| CAPA CO2 | 10 | 1.580 | 1.605 | 0.2858 | 0.09038 | Mann-Whitney-Rank-Sum | 0.0986 |
| EH Air | 12 | 0.4386 | 0.2537 | 0.4719 | 0.1362 |  |  |
| EH CO2 | 10 | 0.3927 | 0.3477 | 0.2131 | 0.06740 | Mann-Whitney-Rank-Sum | 0.0753 |
| **Figure 5. A - Experiment: represents calculated fold changes between Air and CO2 of the respective peptides in previous panels (mean of Air values was substracted from CO2 values). For p-values please see above numbers. N describes number of larvae in CO2 condition. Air condition was calculated with same N numbers:** | | | | | | | |
| DMS | 11 | 1.560 | 1.539 | 0.6962 | 0.2099 |  |  |
| IPCs | 10 | 1.199 | 1.134 | 0.2869 | 0.09073 |  |  |
| DH44 | 10 | 1.664 | 1.604 | 0.7307 | 0.2311 |  |  |
| CRZ | 10 | 1.757 | 1.717 | 0.9676 | 0.3060 |  |  |
| ITP | 11 | 0.8284 | 0.8346 | 0.1990 | 0.05999 |  |  |
| PTTH |  | 0.7804 | 0.7614 | 0.1467 | 0.04639 |  |  |
| HugRG | 10 | 1.077 | 1.051 | 0.1468 | 0.04642 |  |  |
| CAPA | 10 | 0.6776 | 0.6883 | 0.1226 | 0.03877 |  |  |
| EH | 10 | 0.8953 | 0.7927 | 0.4860 | 0.1537 |  |  |
